# Pet Animals and Contagious Diseases

**Published:** 1881-10

**Authors:** 


PET ANIMALS AND CONTAGIOUS DISEASES.—The fact that pet animals can carry contagion, and thus be the means of spreading fatal diseases, is not widely known nor duly appreciated. We have heard of authentic cases in which scarlet fever was communicated from one person to another by means of a cat. Dr. Hewitt, of Lake Superior, relates a somewhat similar instance in which diphtheria was communicated by the same animal. He had noticed for several days that his pet cat was suffering from an enlargement of the glands of the neck; he also remarked the same in other cats. His cat found a resting place in the wall behind the stove, and there died. The day the animal was removed, diphtheria, in its most violent form, broke out in his family, resulting in the death of two or three of his children, and the doctor himself barely escaping with his life. Up to this time the community was remarkably free from sickness of any kind. It was the start of a severe epidemic.

We refer to this subject in hopes that more facts bearing upon it may be communicated by our readers. These are at present scarce, but a little attention paid to the matter would no doubt secure much that would be of importance to comparative and to preventive medicine.

